# Advances in the Application of Exosomes Identification Using Surface-Enhanced Raman Spectroscopy for the Early Detection of Cancers

**DOI:** 10.3389/fbioe.2021.808933

**Published:** 2022-01-11

**Authors:** Lu Yang, Jingyuan Jia, Shenglong Li

**Affiliations:** ^1^ Department of Internal Medicine, Cancer Hospital of Dalian University of Technology (Liaoning Cancer Hospital and Institute), Shenyang, China; ^2^ School of Optoelectronic Engineering and Instrumentation Science, Dalian University of Technology, Dalian, China; ^3^ Department of Bone and Soft Tissue Tumor Surgery, Cancer Hospital of Dalian University of Technology (Liaoning Cancer Hospital and Institute), Shenyang, China

**Keywords:** surface-enhanced Raman spectroscopy (SERS), exosome, early cancer diagnosis, nanoparticles, cancer

## Abstract

Exosomes are small nanoscale vesicles with a double-layered lipid membrane structure secreted by cells, and almost all types of cells can secrete exosomes. Exosomes carry a variety of biologically active contents such as nucleic acids and proteins, and play an important role not only in intercellular information exchange and signal transduction, but also in various pathophysiological processes in the human body. Surface-enhanced Raman Spectroscopy (SERS) uses light to interact with nanostructured materials such as gold and silver to produce a strong surface plasmon resonance effect, which can significantly enhance the Raman signal of molecules adsorbed on the surface of nanostructures to obtain a rich fingerprint of the sample itself or Raman probe molecules with ultra-sensitivity. The unique advantages of SERS, such as non-invasive and high sensitivity, good selectivity, fast analysis speed, and low water interference, make it a promising technology for life science and clinical testing applications. In this paper, we briefly introduce exosomes and the current main detection methods. We also describe the basic principles of SERS and the progress of the application of unlabeled and labeled SERS in exosome detection. This paper also summarizes the value of SERS-based exosome assays for early tumor diagnosis.

## Introduction

The exosome was originally isolated from sheep reticulocytes as a small vesicle with the characteristics of the reticulocyte cytosol (Trams et al., 1981; Pan et al., 1985; Grasso et al., 1990). Researchers only considered it to be a “waste product” of erythrocyte maturation, and as exosomes have been studied more closely, it has been confirmed that exosomes are extracellular vesicles (EVs) secreted by the cell in the form of cytosol (Han et al., 2021b; Clark et al., 2021; Isaac et al., 2021). Exosomes are multi-vesicular endosomes that originate from the endocytic system and are formed through four processes: outgrowth, invagination, multivesicular body formation and secretion, and are spherical, flat or cup-shaped vesicles with a diameter of 30–150 nm (Spilak et al., 2021; Staubach et al., 2021; Sun et al., 2021). In recent years, it has been found that exosomes are distributed in human body fluids, including blood, urine, saliva, thoracoabdominal fluid, cerebrospinal fluid, bile, milk, tears and semen (Yu et al., 2021a; Zhang et al., 2021b; Lin et al., 2021; Mohammadi et al., 2021; Richter and Lehr, 2021). In addition, exosomes of different tissue sources carry different nucleic acids, proteins and lipids (Teng and Fussenegger, 2020; Shehzad et al., 2021; Wu et al., 2021). Exosomes can transmit information and function between cells through the nucleic acids, proteins, and lipids they carry (Xu et al., 2020; Zhu et al., 2020; Azparren-Angulo et al., 2021). Moreover, exosomes play an important role in tumorigenesis and progression, invasive metastasis and immune escape (Hu et al., 2020; Morrissey and Yan, 2020; Thietart and Rautou, 2020; Yan and Jiang, 2020). It can be used not only as a biomarker for tumor diagnosis, but also as a target for tumor therapy (Ariston Gabriel et al., 2020; Dai et al., 2020; Zhou et al., 2020).

As a non-destructive, non-contact and repeatable rapid detection technology, Raman spectroscopy can provide fingerprint information of molecular structure vibration and the peaks are narrow and not easy to overlap. It has significant advantages in the detection of biochemical substances. However, Raman spectroscopy has a cross section of only 10–30 cm^2^/molecule and is susceptible to fluorescence interference (Prieto et al., 2016), which limits the wide application of Raman spectroscopy. In the 1970s, it was discovered that when pyridine was adsorbed on a rough silver surface, a high-intensity Raman spectrum signal could be obtained (Leitch et al., 2012). The Raman effect of Raman spectroscopy is derived from molecular vibrations and rotations, which enables the non-invasive analysis of the composition and structure of substances (Markina et al., 2021; Tanwar et al., 2021; Naqvi et al., 2022). However, conventional Raman spectroscopy is extremely weak, susceptible to fluorescence interference, and has low detection sensitivity, which limits its application. Surface-enhanced Raman spectroscopy (SERS) is a method that uses light to interact with nanostructured materials such as gold and silver to produce a strong surface plasmon resonance effect, which can significantly enhance the Raman signal of molecules adsorbed on the surface of nanostructures to obtain ultra-sensitive fingerprints of the sample itself or Raman probe molecules (Zhang et al., 2021a; Berry et al., 2021; Liu et al., 2021b). SERS has become a promising technique for bio-detection (Wang et al., 2021a; Wang et al., 2021b) by compensating for the shortcomings of conventional Raman spectroscopy with local enhancement of up to 10^14^ in the plasma gap and sharp protrusions. In addition, SERS has a number of advantages. It is highly sensitive and can be used for single molecule detection and resistant to photobleaching and photodegradation. Besides, it is suitable for long-term monitoring (Zhao et al., 2021a; Lopez-Lorente, 2021). SERS technology is widely used in various biomedical fields, such as nucleic acid, protein, human cells, human tissues and human body fluids (Calderon et al., 2021; Ma et al., 2021; Xi and Liang, 2021), and has important applications in the diagnosis of tumors (Guerrini et al., 2021; Shen et al., 2021; Sun et al., 2021). Nowdays, it is believed that physical enhancement mechanism and chemical enhancement mechanism are two explanation mechanisms of SERS enhancement. Among them, physical enhancement is also called electromagnetic enhancement. The interaction of Ag nanostructures will cause the collective oscillation of free electrons on the metal surface. When the frequency of incident light matches the natural oscillation frequency of free electrons in the metal, localized surface plasmon excitation Element Resonance (LSPR) will occur, which leads to the enhancement of the incident light field. Studies have shown that the enhancement factor of electromagnetic field enhancement can reach 10^11^ (Ding et al., 2017). Chemical enhancement is a charge transfer enhancement (Tong et al., 2011; Zhan et al., 2019), which requires the analyte to be directly adsorbed or bonded to the rough metal surface through chemical bonds, and there is a charge transfer with the metal surface. When the energy of the incident photon and electrons are on the metal substrate and the adsorbate When the transfer energy difference between them is equal, resonance will occur, which will increase the effective polarizability of the system and increase the Raman signal. Studies have shown that the chemical enhancement factor is 10^1^–10^3^ (Gruenke et al., 2016). It is generally believed that the SERS effect is the result of the above-mentioned two enhancement mechanisms. At present, a large number of researches on the selection of SERS metal substrates show that Au and Ag nanostructures have good stability, repeatability and easy preparation, It has become the first choice of researchers for SERS substrates, and the anisotropic and sharp morphology nanoparticles have a better SERS enhancement effect than symmetrical nanoparticles (Abdulhalim, 2014).

In recent years, attempts have been made to use the ultra-high-sensitivity SERS technique for exosome detection in an attempt to develop a new technique for efficient and nondestructive liquid biopsy (Chen et al., 2021b; Guerrini et al., 2021). Strategies for exosomes detection based on SERS technology can be divided into two types. One is the unlabeled SERS technique that directly acquires the Raman signal of the exosome itself without the introduction of a Raman probe (Yan et al., 2019; Shin et al., 2020b). The advantage of this method is that it is simple and fast and allows a more direct analysis of the exosome composition. However, the signals of various biomolecules may overlap with each other, resulting in partial loss of diagnostic signal. The other is the labeled SERS technique, which uses Raman probe molecules to label exosomes and indirectly detects the presence and concentration of exosomes by detecting the Raman intensity of the labeled molecules (Shin et al., 2020a; Dong et al., 2020; Fan et al., 2021). In this review, we summarized the application and research of SERS technology in the detection of exosomes, and discussed the advantages and disadvantages of SERS compared with other exosome detection technologies. In addition, we also summarized the research on the detection of exosomes based on SERS in the early diagnosis of tumors, and proposed new insights for future research directions.

## Detection of Exosomes

**FIGURE 1:**
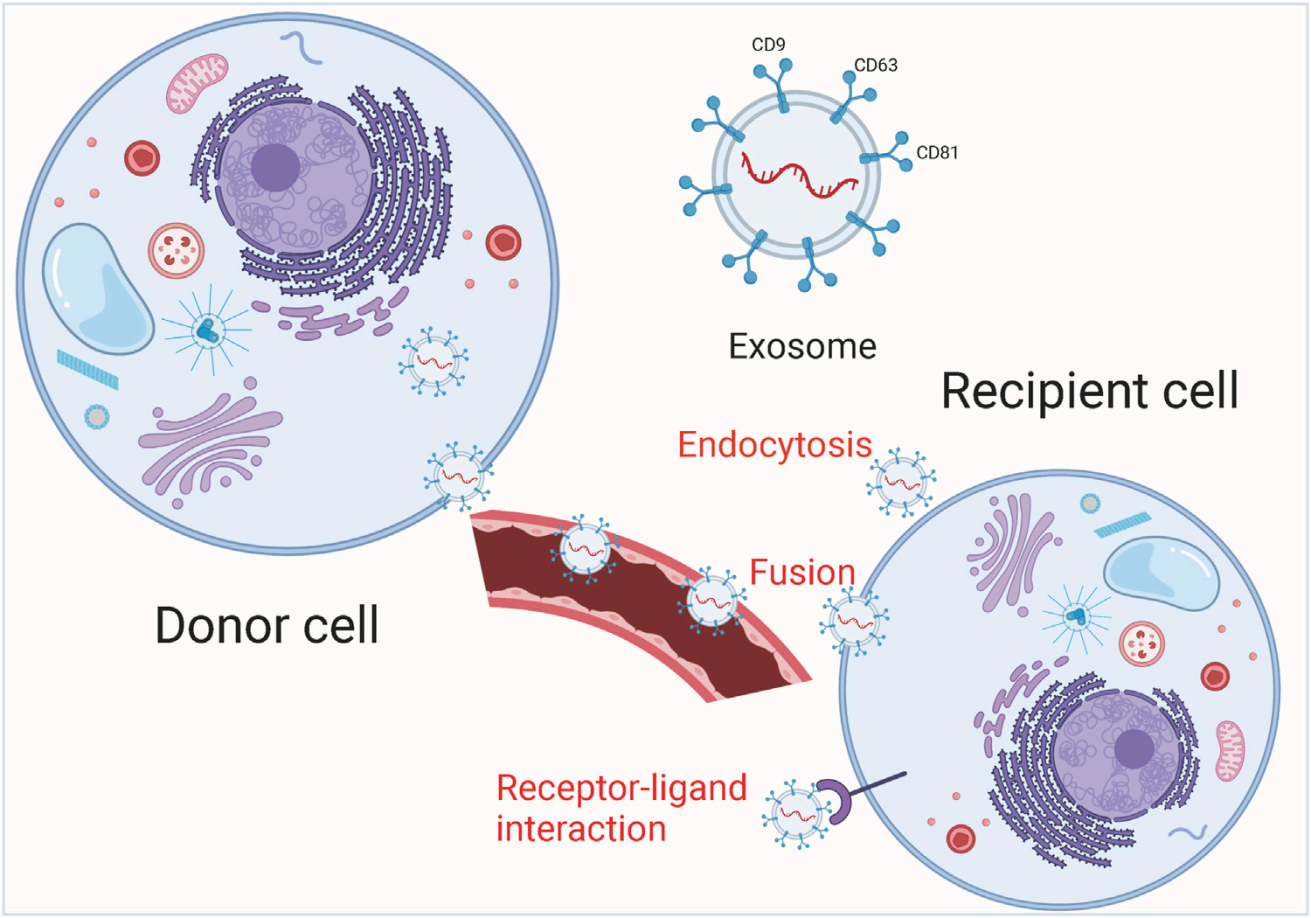
The main process of exosome release. Exosomal cargoes from the source cell can be further delivered to recipient cells *via* endocytosis, direct membrane fusion and receptor-ligand interaction.

Under physiological and pathological conditions, exosomes can be actively secreted extracellularly by various cells including immune cells, stem cells and tumor cells, and can transport a large number of biomolecules from the parent cell to other cells, which is closely related to the pathogenesis of tumors (Claridge et al., 2021; Oey et al., 2021; Yamada, 2021). Because of their small size, exosomes are able to avoid phagocytosis by mononuclear macrophages and cross the vascular wall to the extracellular matrix, they are widely present in various body fluids including blood, saliva, urine, cerebrospinal fluid, and thoracoabdominal fluid (Clark et al., 2021; Leggio et al., 2021; Mao et al., 2021; Prieto-Vila et al., 2021). In addition, exosomes are relatively biologically stable and can be found in tissues and cells due to their phospholipid bilayer structure (Chen et al., 2021a; Hu et al., 2021). Information transfer between exosomes and target cells is achieved through 3 main pathways (Figure 1). Exosomes can enter the recipient cell through receptor-ligand interactions (Isaac et al., 2021; Staubach et al., 2021). Besides, exosomes can also be endocytosed by the recipient cells through phagocytosis (Chen et al., 2021c; Hur and Lee, 2021). Exosomes have an important role in tumor development and can serve as potential diagnostic markers and therapeutic targets for many tumors. In the tumor microenvironment, non-coding RNAs can be distributed in cancer cells, stromal cells and immune cells through small extracellular vesicles (sEVs) and participate in intercellular communication. Sev-nc RNA can be used as a good diagnostic and prognostic fluid marker for tumors (Reese and Dhayat, 2021; Shin et al., 2021; Tang et al., 2021). Exosomal-derived long noncoding RNA (lncRNA) TMZ-associated lnc RNA in GBM recurrence (lnc-TALC) modulates the glioblastoma (GBM) microenvironment and reduces the sensitivity of GBM to temozolomide chemotherapy, targeting exosomal-lnc-TALC may overcome TMZ resistance in GBM (Li et al., 2021b). Lnc RNA colorectal neoplasia differentially expressed (lnc-CRNDE) is highly expressed in tumor-associated macrophages (TAM) of gastric cancer (GC) patients, and CRNDE can be transferred from M2-polarized macrophages to GC cells *via* exosomal form and further promote GC cell proliferation and inhibit cisplatin resistance in GC cells (Xin et al., 2021).

**FIGURE 2:**
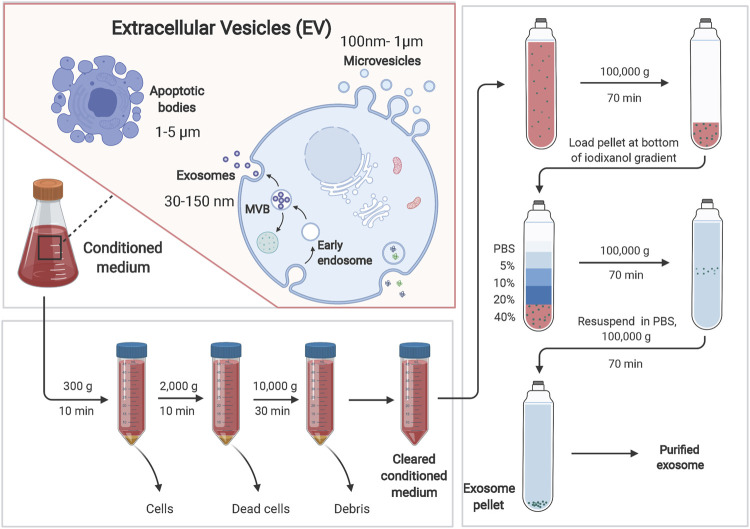
Exosomes can be separated by ultracentrifugation-based isolation techniques. Extracellular vesicles (EVs) can be divided into three main categories: microvesicles, exosomes and apoptotic bodies. Ultracentrifugation-based isolation techniques are the most used for the isolation of exosomes. Brifely, the culture supernatant was centrifuged at 300 × g for 10 min at 4°C, at 2,000 × g for 10 min, and at 10,000 × g for 30 min. After centrifugation, the supernatant was collected. The supernatant was ultra-centrifuged at 100,000 × g for 70 min to pellet the exosomes. Exosomes were washed with PBS and pelleted by ultracentrifugation for 70 min at 100,000 × g.

Exosome-related assays currently include isolation and purification, physical characterization, and analysis for downstream functional studies. The main techniques for the isolation and purification of exosomes include ultracentrifugation, density gradient centrifugation, size exclusion chromatography, ultrafiltration, affinity assay and polymer-based co-precipitation method, while ultracentrifugation-based isolation techniques are the most used for the isolation of exosomes (Figure 2; Table 1) (Ng et al., 2021). Exosomes are detected, isolated and purified in normal or tumor cell culture medium supernatants, blood, saliva and other body fluids (Liu et al., 2021a; Yu et al., 2021c). Physical characterization techniques for exosomes include electron microscopy, dynamic light scattering, flow cytometry and nanoparticle tracking analysis, and tunable resistive pulse sensing assays (Zhao et al., 2021b; Liu et al., 2021c). Techniques for exosomal protein analysis include enzyme linked immunosorbent assay (ELISA), protein blotting and mass spectrometry (Lee et al., 2021; Xu et al., 2021). Nucleic acid analysis techniques include second-generation sequencing, gene chips, and polymerase chain reaction (PCR) (Yu et al., 2021b; Wang et al., 2021c; Zhang et al., 2021c). These analytical techniques have greatly facilitated the research process of exosomes, but there are some limitations: ELISA requires a large number of highly concentrated samples and cannot achieve rapid analysis of complex samples. Nanoparticle tracking analysis techniques have a limited range of measurement of exosome concentrations. Flow cytometry analysis of exosomes may have weak recovery signals; transmission electron microscopy, PCR and tamseq second-generation sequencing techniques are expensive, complicated and time-consuming. In conclusion, there is still no unified standard for exosome analysis and therefore, there is an urgent need to further develop new methods for exosome detection to accelerate the development of cancer body fluid biopsy.

**TABLE 1:** The separation and identification techniques for exosome.

Classification	Detection technology	Aadvantages	Disadvantages
Separation	Ultracentrifugation	Mature method	Need expensive equipment, time-consuming and laborious operation, and easy to interference from protein aggregates and lipoproteins
		suitable for large-volume samples	
	Density gradient centrifugation	The separation purity is improved	The operation is cumbersome and the centrifugation takes a long time
	Size exclusion chromatography	There is no need to use a centrifuge	The purification column has strict limits on the volume of the sample and is not suitable for processing larger samples
		high purity and the method is reproducible	and the eluent will dilute the sample, resulting in a low concentration of the final product
	Ultrafiltration	The separation of exosomes can be achieved at a low speed, which takes a short time and does not affect the biological activity of exosomes	The vesicles and proteins adsorbed by the filter membrane not only seriously affect the extraction efficiency of exosomes, but may also block the filter pores
	Affinity	Good specificity, the only way to identify exosomes derived from specific cells	Time-consuming operation, low extraction efficiency, not suitable for large-scale analysis
	Polymer-based co-precipitation method	Separation can be achieved by conventional centrifugation	The extracted product contains a large amount of lipoprotein and
		simple operation and high recovery rate of vesicles	RNA complexes with very poor purity

## SERS Technology for Exosome Detection

### SERS Technology for Unlabeled Detection of Exosomes

The unlabeled assay is a Raman spectroscopy of exosomes obtained by adsorbing the purified exosomes directly or very close to the SERS substrate surface (Soares Martins et al., 2018; Zhang et al., 2019b). The signal intensity in SERS decays exponentially as the analyte moves away from the hot spot (Liu et al., 2021b; Tahir et al., 2021). To obtain the desired exosome Raman signal, efficient SERS-enhanced substrates need to be prepared. To use unlabeled SERS for exosomes detection, several issues need to be addressed. First, since exosomes are mainly composed of non-chromophore biomolecules, it is difficult to detect their unique signal patterns. Exosomes are larger relative to SERS hotspots, and therefore it is difficult to locate exosomes in large numbers in SERS hotspots. It is important to develop a dedicated exosome detection strategy to acquire their signals sensitively. The Raman spectra of exosomes are heterogeneous and complex, and the molecular fingerprint signals also involve information from many biomolecules. In addition, since exosomes are larger than small biomolecules, identifying the relative position of exosome on the surface of metal nanostructures is challenging for signal intensity. The complex composition of the exosome in body fluids makes the analysis even more difficult. Therefore, effective methods are needed to interpret and analyze complex and heterogeneous signals.

Stremersch et al. (2016) A positively charged gold nanoparticle with enhanced Raman signal was prepared and analyzed using partial least squares discriminant analysis (PLS-DA) with multivariate curve resolution alte RNAting least squares (MCR-ALS) analysis algorithm to analyze the obtained Raman spectral data and be able to distinguish between vesicles from normal and cancer cells. The use of SERS probes decorated with Raman reporter genes and specific aptamer gold nanoparticles for targeting exosomes to capture substrates can be of great benefit, as it can capture most types of exosomes by recognizing the universal surface protein CD63. Based on this, exosomes can be detected in the blood of patients (Wang et al., 2018). The non-tagged SERS technology presents a “fingerprint” of the exosome itself and then uses multivariate statistics and computerized deep learning algorithms to dig deeper into the diagnostic information and build a discriminatory model that can eventually distinguish and trace exosomes from different sources. Currently, there are many difficulties in translating the non-labeled SERS exosome detection technology to the clinical setting, but with the expansion of the number of exosome samples and the development of related technologies, it may be applied to clinical testing in the future. In the non-label detection method, the sample to be tested is directly adsorbed on the surface of the metal nanostructure, and the SERS technology is used to directly obtain the Raman fingerprint of the biological sample itself. This method requires a combination of special nano-substrate structure and spectral analysis technology to distinguish between Spectral information of biomolecules or cells of different species and microorganisms. The advantage of non-label SERS nucleic acid detection technology is that the steps are simple and no special processing is required for samples. However, in general, this method has not yet met the sensitivity of clinical detection and specific requirements.

### SERS Technology for Exosomes Labeling Detection

**FIGURE 3:**
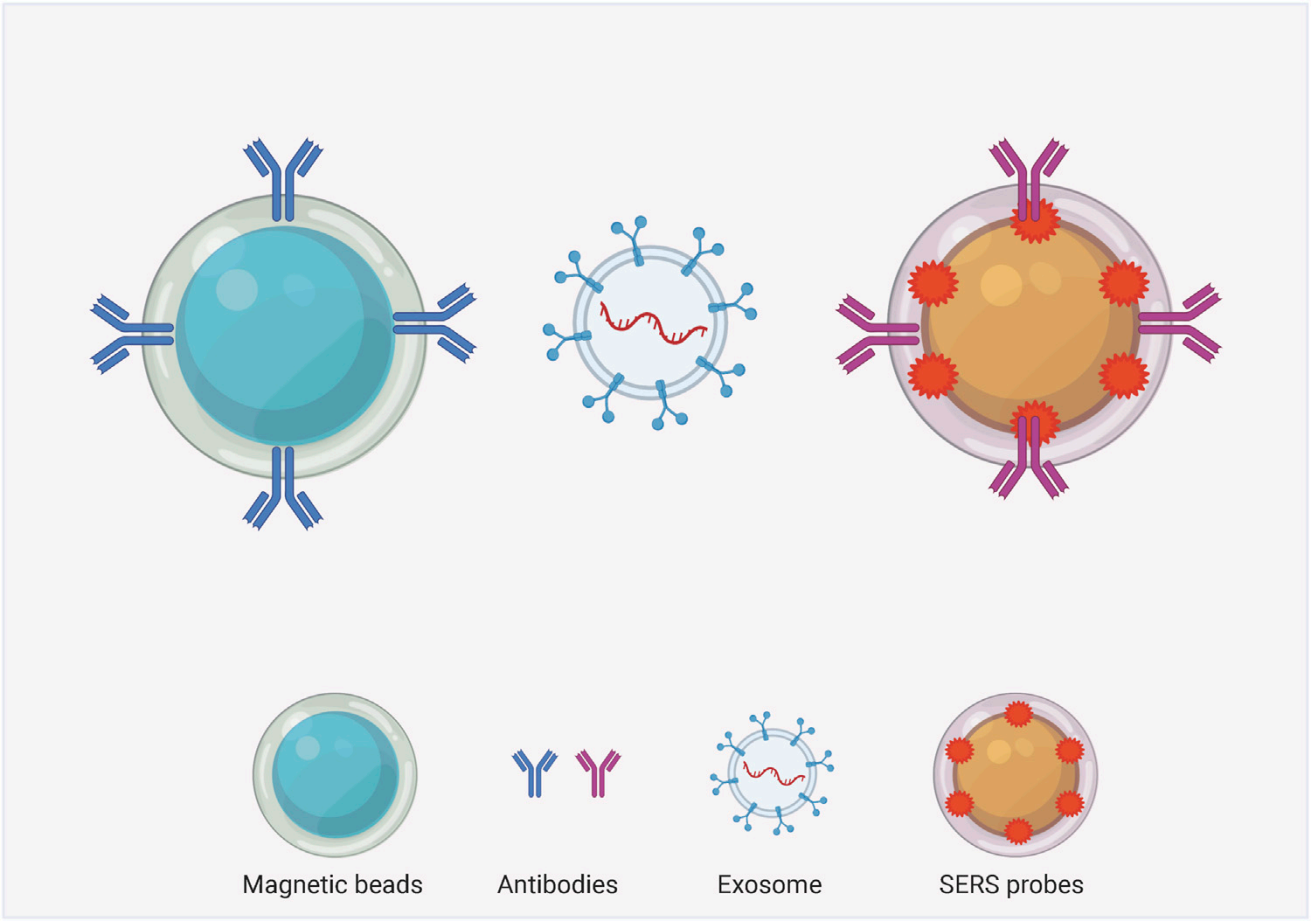
Structural type diagram of exosome-magnetic bead-based SERS assay model.

**FIGURE 4:**
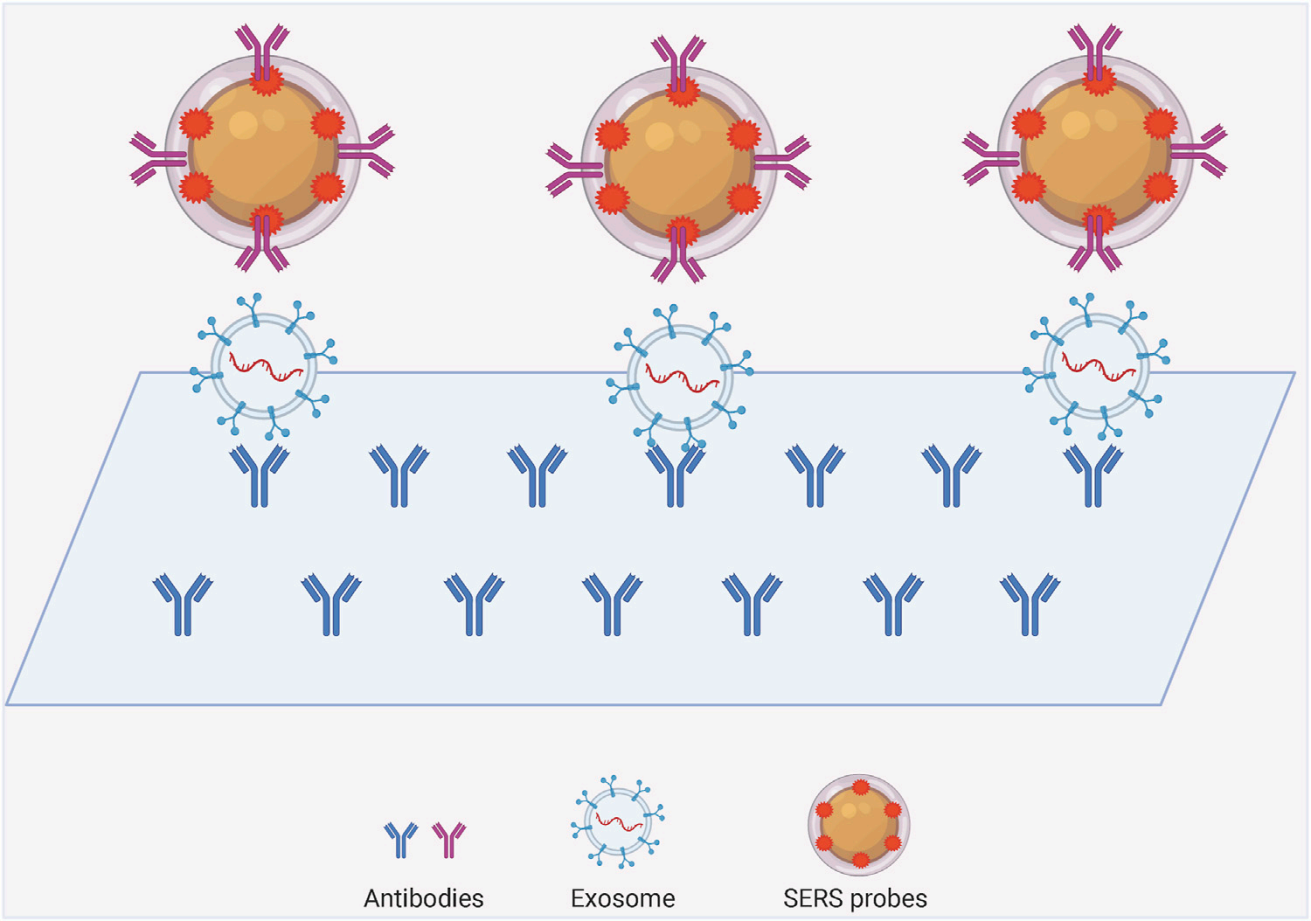
Structural type diagram of exosome-chip-based SERS assay model.

The SERS labeling assay is a nano-labeling immunoassay technique that combines SERS spectroscopy with the specific action of antibody antigens, which are plasma nanoparticles containing a strong Raman signal. The SERS nanomarkers are a class of plasma nanoparticles containing strong Raman signals. The SERS nanomarkers form a complex with the target exosomes through a specific reaction similar to that of antibody antigens, and the distribution and concentration of the target molecules are indirectly characterized by measuring the Raman intensity of the Raman-labeled molecules in the complex. The common structures can be mainly classified into exosome-magnetic bead-based SERS model (Figure 3) and exosome-chip-based SERS model (Figure 4).

### Exosome-Magnetic Bead-Based SERS Assay Model

Compared with normal cells, tumor cells secrete more exosomes and have specific biomarkers on their membrane surface, which makes tumor-derived exosomes an important cancer biomarker. Therefore, an exosome-magnetic bead-based SERS assay model can be designed to take advantage of the fact that tumor-derived exosomes contain a variety of specific proteins. This model typically uses two types of nanoparticles-magnetic nanobeads and SERS nanoprobes, and two specific antibodies are modified on the surface of both particles, which can recognize two different proteins on exosomes separately. In the presence of targeted exosomes, an immune complex can be formed between the SERS nanoprobe, the exosome and the magnetic nanoparticles. In the presence of a magnet, the immunocomplex can precipitate, so the SERS nanoprobe will detect the SERS signal in the precipitation. In contrast, in the absence of targeting exosomes, the SERS signal is barely detectable because no immune complexes can be formed and only magnetic nanoparticles are precipitated. In this way, the SERS signal can be used to discriminate the presence of target exosomes. In recent years, more and more people are discovering the potential of exosome-magnetic bead-based SERS detection models in many studies. In order to identify three different exosomes secreted by cancer cells, researchers prepared capture probes (Wang et al., 2018) by modifying three corresponding aptamers and Raman reporter molecules on the surface of gold nanoparticles. Three probes were present in the supernatant, and when a target exosome was added, it was specifically adsorbed with the corresponding magnetic beads and probes, and the complexes were aggregated to the bottom of the tube under the action of a magnet. At this point, the capture probe for this exosome in the supernatant is significantly reduced, and the corresponding Raman peak for this probe is significantly weakened. In addition, Tian et al. (2018) Similarly demonstrated that the exosome-bead-based SERS assay model has considerable potential as a convenient and highly sensitive quantitative tool for the detection of exosomes in biological samples. Pang et al. (2020) proposed a method that can capture and analyze PD-L1 exosomes directly from serum. Because tio2 shells can bind to the hydrophilic phosphate of phospholipid molecules on exosomes, they used Fe3O4@tio2 nanoparticles to enrich exosomes. PD-L1 exosomes can be quantitatively labeled using PD-L1 antibody-modified Au@Ag@ MBA-SERS labeling. Mi RNA plays an important role in the post-transcriptional regulation of gene expression and plays an essential role in the diagnosis and treatment of tumors (Li et al., 2021a; Andrikopoulou et al., 2021; Crudele et al., 2021; Konoshenko et al., 2021; Oh-Hohenhorst and Lange, 2021). The nature of the exosomal lipid bilayer avoids miRNA degradation, making the specific miRNAs in exosomes well suited as biomarkers for early cancer diagnosis and prognosis. Ma et al. (2018) established a SERS method based on double-stranded specific nuclease-assisted circular amplification for the quantitative detection of miRNAs in human blood exosomes. Pang et al. (2019) validated the feasibility of a SERS biosensor with double-stranded specific nuclease-assisted cycle amplification. This assay further amplifies the signal with nuclease assistance, allows for single base recognition with detection limits, and is suitable for clinical diagnosis without the need for multiple washes and hybridizations (Pang et al., 2019). A Raman scattering probe with a strong electromagnetic hot spot was developed by assembling gold nanoparticles in triangular pyramidal DNA, which can be used for efficient and sensitive detection of circulating tumor cells and exosomes (Zhang et al., 2019a). The MIO structure allows the capture and analysis of exosomes from the plasma of cancer patients without any labeling process. The SERS intensity at 1,087/cm of exosomes caused by P-O bonding within phosphoproteins can be used as a standard for liquid tumor biopsies. The 1,087/cm SERS peak intensity of exosomes extracted from the plasma of cancer patients is at least twice that of healthy individuals (Dong et al., 2020). Chen et al. (2021b) utilized 4-mercaptobenzoic acid-labeled silver nanoparticles attached to the outer surface of exosomes, where the silver nanoparticles can be used as SERS generators. The Raman reagent 4MBA is susceptible to specific intracellular stimuli and can exhibit different vibrational spectral features accordingly. The use of SERS spectroscopy enables the tracking of intracellular distribution of exosomes and simultaneous quantitative sensing of environmental ph (Chen et al., 2021b).

### Exosome-Chip Based SERS Assay Model

Labeled SERS has great potential for qualitative and quantitative studies of exosomes. The method of exosome isolation and enrichment can be done using SERS chips in addition to magnetic beads. Using two different proteins on the exosome and then modifying two specific antibodies on the substrate and probe respectively, an immune complex can be formed between the SERS probe, exosome and the chip in the presence of the targeted exosome, and then the excess non-targeted exosome can be rinsed for SERS detection. Lee and others invented a SERS-based sensing platform for the quantitative determination of exosomal miRNA (Lee et al., 2019). They first modified the capture probe on a homogeneous gold nanopillar substrate, and the target exosomal miRNAs were captured by the probe sequence when they were present. Since the target exosomal miRNAs have long sequences and some sequences are exposed, sequences with Raman reporter molecules are added to complementarily ligate with the exposed sequences to target both the target exosomal miRNAs and the Raman probe on the substrate. SERS detection probes were made using bimetallic SERS active nanotags, gold-silver-silver core-shell-shell nano sea cucumbers (gssnts), decorated with junctional DNA, complementary to the aptamer of the targeted exosome. In the presence of exosomes, the aptamer specifically recognizes and captures the exosomes and subsequently releases the gssnts into the supernatant. The presence of the target exosome is indicated by the detection of an attenuated SERS signal on the MB (Ning et al., 2020). A densely packed and ordered Au octahedral array was used as a sensing platform for the quantitative determination of let-7a in MCF-7 cell-derived exosomes. Where the gold octahedra in the array stand uniformly on their triangular faces, significantly improving detection sensitivity and uniformity. This model enables let-7a detection with single-base specificity in the presence of a target by detecting the SERS intensity change caused by the conversion of DNA from a hairpin structure to a double-stranded body (Kang et al., 2021). Chen et al. (2020) A novel exosome/metal nanohybrid was constructed. The heterogeneous exosomes can be internalized by lattice-protein-mediated endocytosis and then translocated to lysosomes. The metal nanoparticles in the hybrid were shown to have no effect on exosome features while serving as a surface-enhanced Raman scattering generator. This technique may improve the utility of exosome nanohybrids in therapeutics such as multifunctional drug delivery systems (Chen et al., 2020).

### The Sensitivity of SERS in Detecting Cancer-Derived Exosomal Protein and Nucleic Acid

Protein is one of the vital biological macromolecules in living organisms, and is the main bearer of life activities in energy storage, metabolism, and cell function regulation. Abnormally expressed exosomal proteins usually appear in the early or late stages of tumors. It has important diagnostic significance in its occurrence and progress. Therefore, sensitive, accurate and specific quantitative analysis is of great significance. SERS has been proven to have great potential in high-throughput biomolecular detection, including protein Detection. The research on protein detection by SERS only involves the side chains of amino acids, and some studies have obtained signals from the two amide groups and side chains. The inte RNAl structure information of biomolecules (non-labeled method) and the exte RNAl SERS label (labeled method) are two independent methods for direct and indirect detection of biomolecules. The labeling method requires an appropriate dye label. If the label has resonance under a certain excitation line, it can provide an additional enhanced signal, so the detection sensitivity of the labeling method is higher. Instead of labeling, all the information obtained is the amino acid skeleton of the protein. The reproducibility of the spectrum is poor, the non-reproducibility of protein adsorption sites and the denaturation of protein adsorption on the metal surface will all be studied for non-labeling methods. Bring difficulties. However, the information given by non-labeled means can analyze protein structure more directly, which is of great help to protein analysis.

Nucleic acid is a biological macromolecular compound formed by nucleotide as a synthetic unit. It is the most basic genetic material of life and plays a decisive role in a series of major life phenomena such as growth, inheritance, and evolution of life. Carrying out nucleic acid molecular diagnosis is of great significance to promoting the development of human health and medical treatment. SERS has unique technical advantages in the detection and analysis of biological samples such as nucleic acids. First of all, SERS has low requirements on the shape and size of the sample to be tested, which can save complicated sample pretreatment steps. Secondly, SERS has a small sample requirement and is suitable for the detection of trace and trace samples. In addition, SERS can achieve rapid detection, generally within 30 s to complete the detection of a sample. The introduction of Raman labeling technology can achieve the purpose of strong specific detection. Moreover, because the Raman scattering of water is very weak, the Raman spectroscopy technique is very suitable for the detection of samples in the water environment. SERS can also be combined with other detection methods to increase detection sensitivity and specificity. SERS combined with PCR nucleic acid detection method can successfully realize the recombination and concentration amplification of the target sequence, so as to achieve the purpose of ultra-sensitive detection. The nucleic acid SERS detection based on enzyme digestion technology mainly relies on the excellent SERS enhancement performance of the “hot spots” between metal nanoparticles, uses the self-assembly of ss-DNA to increase the probability of hot spots formation, and uses enzyme digestion reactions to eliminate hot spots to achieve SERS signals. Change from weak to strong and then from strong to weak.

However, there is no research report comparing SERS and other traditional detection methods in the detection of exosomal-derived proteins or nucleic acids. Whether SERS has outstanding advantages in the detection of exosomal-derived proteins or nucleic acids still needs further research.

### The Value of SERS-Based Exosome Detection Technology in Early Tumor Diagnosis

Exosomes are promising biomarkers present in body fluids and cell lines in cancers. As described above, plenty of SERS-based exosomes have been proposed in combination with specific recognition/separation/amplification techniques for qualitative/quantitative analysis of individual targets (Table 2).

**TABLE 2:** Cancer-derived exosomal biomarkers detected by SERS-based techniques.

Cancer type	Sample type	Detection method	Detection results	Reference
Ovarian cancer	Exosomes secreted by SKOV3 cells	Silver film-coated nanobowl platform, label-free SERS detection, PCA analysis	High presence of RNA, compared SERS spectra from UC-purified exosomes and TIER-purified exosomes, monitored changes of SERS spectra	Lee et al. (2015)
Lung cancer	Exosomes secreted by lung normal and cancer cell	Au NPs on cover glass, label-free SERS detection	Distinguished from normal cell-derived exosomes by 95.3% sensitivity and 97.3% specificity	Park et al. (2017)
		PCA analysis		
	Exosomes secreted by lung normal, cancer cell and patients’ plasma	A GNP colloidal solution onto a 3-aminopropyltriethoxysilane (APTES)-coated coverslip, label-free SERS detection	Identified early-stage lung cancer patients with high accuracy	Shin et al. (2020a)
		PCA-linear discriminant analysis (LDA) and partial least-squares discriminant analysis (PLSDA)	The deep learning model supervised by cellular exosomes successfully identified the lung cancer patients and even detected stage I patients	
	Exosomes secreted by lung normal and cancer cell	an aggregated gold nanoparticles (GNPs) substrate was fabricated to form nanogaps, PCA analysis	Contributed to studies on exosomal surface protein markers for diagnosis of cancers	Shin et al. (2018)
	Exosomes secreted by lung cancer patients’ serum (4 μL)	anti-PD-L1 antibody modified Au@Ag@MBA SERS tags	Developed a straightforward and rapid procedure for exosomes isolation and exosomal PD-L1 biomarkers quantification from clinic serum samples	Pang et al. (2020)
	Exosomes secreted by lung cancer patients’ plasma (5 μL)	a new SERS analysis strategy combining stable SERS reporter element and duplex-specific nuclease (DSN)-assisted signal amplification, point-of-care testing (POCT)	With the aid of DSN, one target exosomal miRNA can trigger the release of numerous signal reporter elements, and thus ultrahigh sensitivity could be achieved	Ma et al. (2018)
			The separate effect of SiMB and the stable Raman signatures of ARANPs can ensure the capability of applying this new approach for detection of exosomal miRNA	
Breast cancer	Exosomes secreted by HEK-293T cell	A novel Raman probe (TP-Au NP probe) was developed through the electrostatic attraction between the negatively charged DNA tetrahedron and the positively charged AuNPs	Distinguished exosomes extracted from the plasma of healthy individuals and breast cancer patients	Zhang et al. (2019a)
	MCF-7 cell and patients’ plasma			
	Exosomes derived from MDA-MB-231 cells, MDA-MB-468 cells, SKBR3 cells and MCF12A cells	Au array-EpCAM, CD44, HER2, EGFR, IGF1R, CD81, CD63 and CD9 antibodies, in combination with Au	Identified HER2 and EpCAM biomarkers on exosomes in plasma from HER2-positive breast cancer patients	Kwizera et al. (2018)
		NR-QSY21		
	Exosomes derived from breast cancer cells	A plasmonic gold nanopillar SERS substrate (3 × 3 mm) by maskless reactive ion etching (RIE)	Can be used for universal cancer diagnosis and further biomedical applications through the quantitative measurement of exosomal miRNAs in bodily fluids	Lee et al. (2019)
Pancreatic cancer	Exosomes secreted by pancreatic normal and cancer cell	SERS, label-free SERS detection, principal component and differential function analyses	Exhibited up to 87 and 90% predictive accuracy for healthy control and pancreatic cancer individual samples	Carmicheal et al. (2019)
	Exosomes secreted by pancreatic ductal adenocarcinoma, chronic pancreatitis, normal controls plasma	Fe3O4 @Ag-DNA-Au@Ag@DTNB (SERS tag) conjugates, label-free SERS detection	MicroRNA-10b in the blood samples even can distinguish pancreatic cancer from chronic pancreatitis and normal controls	Pang et al. (2019)
	Exosomes secreted by pancreatic cancer patients’ serum (4 μL)	Locked nucleic acid (LNA)-modified Au@DTNB, Fe3O4@TiO2 nanoparticle	Using exosomal miRNA-10b as a proof of concept, pancreatic ductal adenocarcinoma patients can be recognized from normal controls (NCs) with an accuracy of 99.6%	Jiang et al. (2021b)
Melanoma	Exosomes from RBC and B16F10 melanoma cancer cells	Negatively-charged exosomes functionalized with positively-charged 10 nm Au NPs on their surface; label-free SERS detection and PLS-DA analysis	Exosomes from different origin can be distinguished, even when present in the same mixture	Fraire et al. (2019)
Osteosarcoma	Exosomes derived from osteosarcoma patients and negative controls	SERS and matrix-assisted laser desorption/ionization time-of-flight mass spectrometry (MALDI-TOF MS)	The non-invasive liquid biopsy method using SERS and MALDI-TOF MS	Han et al. (2021c)
			fingerprinting of exosomes has great potential for rapid	
			diagnosis of osteosarcoma	

Ovarian cancer is the most lethal gynecologic malignancy with a 5-years survival rate of only 47.4%, seriously endangering women’s physical and mental health (Baert et al., 2021; Wang et al., 2021d). A silver film coated on the surface of 3D nanobowls as a SERS substrate was used to detect the SERS signal of exosomes of ovarian cancer cells over time using sputtering technique (Lee et al., 2015). In contrast to silver nanoparticles prepared by reduction method, sputtering iso-expressed ovarian cancer exosomes were specifically captured. In addition, this method allows selective detection and analysis of target exosomes from exosome-rich body fluids, a technique with great potential for early disease detection.

Lung cancer has developed into one of the most common malignant tumors in the world, with the highest incidence and mortality rate (Jiang et al., 2021a; Drusbosky et al., 2021). The incidence of lung cancer has been on the rise due to factors such as smoking and environmental pollution (Koch et al., 2021; Raguraman et al., 2021). Developed a label-free, highly sensitive method for exosome classification by combining SERS technology with statistical algorithms (Park et al., 2017). They used gold nanoparticles as an enhanced substrate for SERS and used principal component analysis to analyze the spectra of exosomes secreted by lung cancer cells and normal cells separately and found a sensitivity of 95.3% and specificity of 97.3%. Shin and others used computerized deep learning to perform SERS signals of exosomes from normal and lung cancer cell lines trained and modeled after extracting features of both types of exosome spectra for analysis and prediction of SERS spectra of human plasma-derived exosomes (Shin et al., 2020a). This trial combined SERS spectral analysis of exosomes with deep learning to successfully identify lung cancer patients, even those in stage 1. The method is noninvasive, safe, sensitive, and important for the development of early liquid biopsy methods for lung cancer. Shin and others demonstrated correlation between non-small cell lung cancer (NSCLC) cell-derived exosomes and potential protein markers for cancer diagnosis by unique Raman scattering spectroscopy and principal component analysis (PCA). Based on the SERS signals of exosomes from NSCLC cells and normal cells, they extracted the Raman patterns of cancerous exosomes by PCA and clarified the specific patterns into unique peaks by quantitative analysis of the proportional mixture of cancerous and normal exosomes. Subsequently, they compared these unique peaks with the characteristic Raman bands of several exosomal protein markers. The correlation was used for the diagnosis of NSCLC by analysis (Shin et al., 2018). Pang et al. (2020) Enrichment of exosomes by binding of tio2 shells and hydrophilic phosphate heads of exosomal phospholipids was performed using Fe_3_O_4_@tio2 nanoparticles. Exosomal PD-L1 was labeled and quantified by adding anti-PD-L1 antibody-modified Au@Ag@MBA SERS tags. Based on personalized SERS signal analysis, it was possible to differentiate NSCLC patients from healthy controls and the potential efficacy of immunotherapy (Pang et al., 2020). Preparation of SERS signal reporter molecules with Au@R6G@agau nanoparticles with small nanogaps produces stable SERS signals. Covalent attachment of isolated substrates of ARANP and silica beads to the 3′- and 5′-termini of capture probes targeting exosomal miRNAs specifically recognizes exosomal miRNAs. This technique was used to detect exosomal miRNAs in the plasma of NSCLC cancer patients (Ma et al., 2018).

Breast cancer is a malignant tumor that occurs in the epithelial cells of the breast and is highly heterogeneous in time and space, and its extremely high incidence poses a serious threat to women’s physical and mental health and life safety (Boix-Montesinos et al., 2021; Burstein et al., 2021). Zhang developed an assembling gold nanoparticles in triangular pyramid DNA (TP-Au nps) based on the principle of electrostatic attraction between negatively charged DNA tetrahedra and positively charged Au nps (Zhang et al., 2019a). This sensor with a strong electromagnetic hot spot can greatly enhance the SERS signal. The epithelial cell adhesion molecule content on human breast cancer cell MCF-7 exosomes is high, so epcam aptamers and cholesterol-modified DNA can be selected for exosome identification and enrichment when detecting exosomes. Streptavidin-modified magnetic beads react with biotin-modified epcam aptamers to capture enriched exosomes. The cholesterol-modified DNA hybridizes with TP-Au nps SERS probes and the exosomes are identified by hydrophobic interactions between the cholesterol attached to the SERS probes and the exosomal lipid membrane. This method can effectively distinguish between exosomes extracted from plasma of healthy individuals and breast cancer patients. Circulating exosomal PD-L1 can be used as a predictor of clinical efficacy of anti-PD-1/PD-L1. A template array was immobilized on a standard gold-plated glass lens and then a miniaturized affinity-based device for exosome capture was prepared by modifying antibodies on the array well plate (Kwizera et al., 2018). Gold nanorods bind to exosomes *via* electrostatic interactions between cetyltrimethylammonium bromide and lipid membranes on exosomes, enabling quantitative detection of several surface protein markers on exosomes derived from breast cancer cell cultures and human epidermal growth factor receptor 2 (HER2)-positive breast cancer patients. HER2-positive Exosomes from plasma of breast cancer patients have more significant levels of HER2 and epcam than those from healthy donors, suggesting the diagnostic potential of these markers for HER2 and epcam in breast cancer diagnosis. Lee et al. (2019) developed a SERS-based sensing platform for the quantitative determination of exosomal miRNA. They obtained ultrasensitive exosomal miRNAs with single nucleotide specificity in an enhanced SERS signal from a homogeneous plasma head flocked gold nanopillar substrate. The proposed SERS sensor showed extremely low detection limits without any amplification process, a wide dynamic range and good multiplex sensing capability. Using this model they could analyze the differences in miRNA expression in plasma exosomes of breast cancer patients and differentiate breast cancer subtypes based on the results (Lee et al., 2019).

Pancreatic cancer is a highly aggressive and malignant malignancy of the gastrointestinal tract, and its incidence is increasing year by year (Hou et al., 2021; Huang et al., 2021; Tao et al., 2021). SERS with polydopamine-modified immunocapture substrate and ultrathin polydopamine-encapsulated antibody-reporter gene-Ag (shell)-Au (nucleus) multilayer (PEARL) was developed. Migration inhibitory factor (MIF) antibody-based SERS immunoassay can distinguish not only between pancreatic cancer patients and healthy controls, but also between metastatic and non-metastatic tumors, as well as tumor lymph node metastasis carmicheal used the unlabeled SERS technique in combination with a principal component differential function analysis algorithm to differentiate between exosomes derived from pancreatic cancer and normal pancreatic epithelial cells with 90% discriminatory accuracy. They further applied this method to the analysis of exosomes in serum and also obtained satisfactory results, which is expected to develop a new method for early detection of pancreatic cancer (Carmicheal et al., 2019). A DSN-assisted dual SERS biosensor was developed for the detection of miRNA in exosomes and blood sample residual plasma based on Fe_3_O_4_ @Ag-dnaau@Ag@DTNB (SERS tag) affixes. Early diagnosis of patients can be made by detecting differences in SERS signals in plasma-derived exosomes and residual supernatant plasma from pancreatic ductal adenocarcinoma (PDAC), chronic pancreatitis and normal control blood samples (Pang et al., 2019). Jiang et al. (2021b) Designed an *in situ* platform for direct extraction of exosomal miRNA from serum samples. They synthesized locking nucleic acid-modified Au@DTNB as a SERS tag into exosomes and assembled with target miRNAs to induce hotspot SERS signals. The exosomes were then enriched by the addition of Fe3O4@tio2 nanoparticles for further SERS detection. Using this technique to detect exosomal miRNA-10b can be used to effectively identify patients with PDAC (Jiang et al., 2021b).

Melanoma is a highly aggressive malignancy originating from melanocytes, the incidence of which is increasing every year (Han et al., 2021a; Dimitriou et al., 2021; Worrede et al., 2021). Fraire et al. (2019) demonstrated that a new strategy using core-shell plasma nanoparticles as SERS substrates showed higher near-field enhancement than previous methods, which significantly increased the signal-to-noise ratio of SERS spectra. Individual vesicles of melanoma cells and erythrocytes can be distinguished using this technique (Fraire et al., 2019).

Osteosarcoma is the most common primary malignant bone tumor that occurs in the epiphysis of long bones, mainly in the distal femur, proximal tibia and humerus (Lin et al., 2017; Lu et al., 2020). Approximately 18% of patients with osteosarcoma already have micrometastases at the time of diagnosis (Ballatori and Hinds, 2016). For patients with osteosarcoma who develop metastases and recurrence, their 5-years survival rate remains at about 20% (Gill et al., 2013). Han et al. (2021c) analyzed the potential of SERS and matrix-assisted laser desorption/ionization time-of-flight mass spectrometry (MALDI-TOF MS) for the detection of osteosarcoma. They characterized plasma-derived exosomes from osteosarcoma patients and healthy individuals using SERS and MALDI-TOF MS, and further combined the two types of spectroscopic techniques using a data fusion approach, which revealed through analysis that non-invasive liquid biopsy methods of SERS and MALDI-TOF MS fingerprint mapping of exosomes have great potential for rapid diagnosis of osteosarcoma (Han et al., 2021c).

## Future Perspections and Discussion

Exosomes are found in a variety of body fluids, carry characteristic markers and play a wide range of functions (Han et al., 2021b; Cheng et al., 2021; Peng et al., 2021). The study of exosomes has great potential for new diagnostic and medical translational applications, yet no significant breakthroughs have been achieved to date. Our understanding of the molecular mechanisms of exosomes development and sorting of contents is still in its infancy, and the standardization of exosomes collection and detection techniques for clinical samples is still immature, both of which have limited the development of exosomesas clinically available diagnostic and therapeutic tools. The precise molecular mechanisms of exosomes, the innovation of isolation and purification techniques, and the multi-omics analysis of exosomes are the future directions of exosomes research.

There are still many challenges and limitations in using Raman spectroscopy to analyze biological samples. Higher sensitivity techniques such as SERS can be used when the Raman spectral signal of the sample is weak, but sample preparation plays an important role. When analyzing Raman spectra of biological samples, the complexity of the composition requires a reliable calibration model to ensure reliable and quantitative measurements. There are also challenges when using probes for *in vivo* analysis, where deficiencies such as background fluorescence, artifacts and weak signals need to be overcome and diagnostic models need to be constructed by multivariate statistical analysis methods before introducing Raman spectroscopy as a basis for diagnosis. Raman spectroscopy can analyze changes in diseases at the molecular level, providing objective and quantifiable information for disease diagnosis and evaluation of treatment efficacy. The use of Raman spectroscopy provides more insight into the infiltration process of tumors and the chemical information obtained allows quantitative and qualitative analysis of the disease. Interference such as fluorescence poses great difficulties in acquiring and resolving Raman spectra of biological samples, so there is still a need to develop techniques for acquiring, processing and classifying data to determine the nature of tumor tissue lesions by pre-processing the raw data, eliminating unwanted signals and enhancing Raman spectral features to achieve qualitative and quantitative analysis of the data. With the continuous improvement of Raman spectroscopy database, tissue classification methods and instrument design, it will become possible to acquire Raman spectroscopy data with higher resolution and accuracy and shorter acquisition time, thus enabling early cancer diagnosis. The reproducibility, reliability and sensitivity of SERS biological detection technology depend on the reasonable design of the SERS substrate, the proper handling of biological samples, the precise control of the detection conditions and the adequate analysis of the spectral data. In the future, efforts should be made to control its potential factors. We should prepare SERS substrates with high uniformity, high enhancement effect and clean surface, which can be modified appropriately. We can also choose appropriate Raman probe molecules to improve their biocompatibility, avoid non-specific adsorption, and to be tested In addition, when storing and pretreating biological samples, keep them in an environment close to the normal physiological state as much as possible to avoid irreversible changes. Moreover, select appropriate SERS detection conditions to obtain reliable detection information. More importantly, establish a standard SERS database of biomolecules to accurately identify spectral information. If necessary, the probe response in the detection system can be corrected, or appropriate data processing and statistical analysis methods can be selected. Future SERS technology The prospect in the field of early detection of tumor biomarkers lies in the development of an ultra-sensitive, highly specific, high-throughput and universal detection platform to meet the needs of life sciences and clinical diagnosis.

In recent years, SERS technology has been applied to the study of exosome detection, which has greatly improved the sensitivity of exosome detection, which is significant for the development of new liquid biopsy techniques based on exosomes (Lee et al., 2020; Han et al., 2021c; Das et al., 2021; Fan et al., 2021; Guerrini et al., 2021). The forms of exosome detection by SERS technology are mainly divided into non-labeled and labeled assays. Non-labeled assays provide overall biochemical information about the exosome itself and are easy, fast and inexpensive to perform (Russo et al., 2020; Jiang et al., 2021b; Guerrini et al., 2021). Marker assays, on the other hand, improve the specificity of exosome identification and can also identify and detect secreted exosomes from multiple cancer cell lines simultaneously (Blanco-Formoso and Alvarez-Puebla, 2020; Chen et al., 2020; Hao et al., 2020). Although some progress has been made in exosome detection with SERS technology, many challenges remain for further research. First, we need to continue to explore efficient SERS detection substrates to further improve the Raman signal of exosomes and enhance the detection limit. Secondly, we should continue to develop SERS detection strategies for exosomal surface proteins and internal nucleic acids to obtain high quality and reproducible Raman signals and to extract comprehensive diagnostic information. Moreover, it is important to develop efficient machine learning algorithms for deep mining of exosomal SERS signals to achieve rapid and accurate identification of exosomes from different sources. In the future, we should also explore the functionalization of exosomes and characterize the biological behavior of exosomes in combination with SERS technology. For clinical applications, we should also continue to expand clinical testing samples to further validate the feasibility and applicability of SERS-based exosome detection technology. With further development of related technologies, we believe that SERS-based exosome detection technology is expected to become a new method for nondestructive, convenient, and accurate liquid biopsy.

## Conclusion

We reviewed the latest research and progress in the identification of exosomes by SERS in the early diagnosis of tumors. First, we discussed the current research status of SERS and the main detection methods of exosomes. Then, we summarized the exosomes detection strategies used to obtain the exosomes and unlabeled SERS signals, and classified and summarized the detection strategies according to how to capture exosomes on the SERS substrate. At the same time, we summarized the advantages and disadvantages of SERS in detecting proteins and nucleic acids. Moreover, we analyzed and summarized the research and progress of SERS identification of exosomes in the early diagnosis of different tumors, and proposed possible future research directions based on the current research status. With the further development of related technologies, we believe that the exosomes detection technology based on SERS technology is expected to become a new non-destructive, convenient and accurate liquid biopsy method for tumors.

## Abbreviations

ELISA, enzyme linked immunosorbent assay; EVs, extracellular vesicles; GC, gastric cancer; GBM, glioblastoma; HER2, human epidermal growth factor receptor 2; lncRNA, long noncoding RNA; MCR-ALS, multivariate curve resolution alte RNAting least squares; MIF, Migration inhibitory factor; NSCLC, non-small cell lung cancer; PDAC, pancreatic ductal adenocarcinoma; PCA, principal component analysis; PLS-DA, partial least squares discriminant analysis; PCR, Polymerase chain reaction; sEVs, small extracellular vesicles; SERS, Surface-enhanced Raman spectroscopy; TAM, tumor-associated macrophages.
